# Improved multi-objective differential evolution algorithm based on a decomposition strategy for multi-objective optimization problems

**DOI:** 10.1038/s41598-022-25440-7

**Published:** 2022-12-07

**Authors:** Mingwei Fan, Jianhong Chen, Zuanjia Xie, Haibin Ouyang, Steven Li, Liqun Gao

**Affiliations:** 1 School of Mechanical and Electric Engineering, Guangzhou University, Guangzhou, 510006 China; 2 Guangzhou Key Laboratory of Condition Monitoring and Control of Mechanical and Electrical Equipment, Guangzhou, China; 3 Graduate School of Business and Law, RMIT University, Melbourne, 3000 Australia; 4 College of Information Science and Engineering, Northeastern University, Shenyang, 110004 China

**Keywords:** Engineering, Mathematics and computing

## Abstract

Many real-world engineering problems need to balance different objectives and can be formatted as multi-objective optimization problem. An effective multi-objective algorithm can achieve a set of optimal solutions that can make a tradeoff between different objectives, which is valuable to further explore and design. In this paper, an improved multi-objective differential evolution algorithm (MOEA/D/DEM) based on a decomposition strategy is proposed to improve the performance of differential evolution algorithm for practical multi-objective nutrition decision problems. Firstly, considering the neighborhood characteristic, a neighbor intimacy factor is designed in the search process for enhancing the diversity of the population, then a new Gaussian mutation strategy with variable step size is proposed to reduce the probability of escaping local optimum area and improve the local search ability. Finally, the proposed algorithm is tested by classic test problems (DTLZ1-7 and WFG1-9) and applied to the multi-objective nutrition decision problems, compared to the other reported multi-objective algorithms, the proposed algorithm has a better search capability and obtained competitive results.

## Introduction

The innovation of science and technology and the development of high-quality life force people to pursue more than a single goal and different goals are constantly taken into account and balanced. For example, when customers purchase a product online, they may consider product quality, cost as well as the transportation time instead of only cost. In the process of combination arrangements for the industrial automation production system, both the production efficiency and the quality should be considered in addition to the rational utilization of product material resources. For multi-robot cooperative control, the control stability, accuracy and rapidity should be balanced. More and more real-world multi-objective optimization problems include multiple objective such as stable matching problems^1^, the optimal dispatch of hydropower stations^2^, and power system economic load dispatch are value to explore and solve. Generally speaking, the sub-goals in a multi-objective problem can be contradictory to each other, and the improvement of one sub-goal may result in the performance degradation of another or several other sub-goals. That is, it is not possible for several sub-goals to reach the optimal value at the same time, but only for the sub-goals to be reconciled or compromised to achieve the optimal value as much as possible. Therefore, real-world multi-objective optimization problem has complex interleaving and contradiction characteristic and it difficult to solve, which is value to research and analysis.

According to the relationship between decision makers and algorithm searches, the traditional methods used to solve multi-objective optimization problems can be divided into three categories^3^: (1) The policy maker sets an expected goal or preference value for each goal before optimizing. Examples in this category include global criterion method, min–max optimum, and goal programming method. (2) Optimization takes precedence over decision-making. Instead of taking into account the preference of the decision maker for each objective, a typical method in this category, such as linear combination of weight and ε-constraint method, directly obtains the Pareto optimal solution set through algorithm optimization. (3) Interaction exists between decision-making and optimization. First, a non-inferior solution is obtained, and the preference value for the objective function is then adjusted based on the judgment of policy maker. The process is repeated until the result is satisfactory or the result can no longer be optimized. Examples in this category include STEP and PROTRADE, etc. The above three categories of traditional methods can solve some multi-objective optimization problems quickly and effectively. However, for the first and third categories, the preference values of the decision makers for the objectives must be obtained, otherwise the algorithm will not get the correct optimization direction. It should also be noted that the traditional methods often incur extensive computation time obtain the Pareto optimal solution set.

The aforementioned traditional methods can solve simple MOP efficiently. However, decision makers using the first or third method must set the preference to each object before or after optimization, or the optimization will be futile because algorithms cannot find the correct optimizing direction. Besides, the cost of time and calculation is tremendous in these methods. So traditional methods cannot perform well as the problems become more complex.

As the development of heuristic algorithms, many researchers try to apply heuristic algorithms to solve MOP, including nondominated sorting genetic algorithm II (NSGA-II) based on Pareto dominance^4^, Differential evolution algorithm (DE)^5^, multi-objective particle swarm optimization (MOPSO)^6^ and so on. These algorithms mainly employed some new multi-objective handle techniques and competitive swarm intelligent optimization algorithms to search for better pareto front (PF). Various Multi-objective Optimization Evolutionary Algorithms (MOEAs) are available. In the past decades, more and more scholars have paid much attention to MOEAs^7^. In 2001, Abbass et al. first applied differential evolution algorithm to MOP in^8^, in which new population is generated by mutation and crossover operation then nondominated solutions are saved by selecting. The results show that PDE performs better than many algorithms in solving MOP due to the introduction of pareto domination. In 2002, Madavan et al. proposed Pareto Differential Evolution Approach (PDEA) which has two versions in^9^. Though the offspring of both versions are generated with the operation in DE, the generated solutions are decided to be preserved or not by comparing with their main parents in the first version, while in the other version the new population is selected by using nondominated and diversity sorting strategy from individuals combining parents and offspring. The latter version is more effective because it has a higher convergence speed. In 2003, Xue et al. proposed multi-objective differential evolution algorithm (MODE)^10^, in which whether the individuals are saved or deleted depends on the fitness based on the nondominated rank and crowded distance. In 2005, a differential evolution for multi-objective optimization (DEMO) which generates new population according to the dominance relationship of parents and offspring is proposed by Jamova et al. in^11^. If the population size is bigger than the setting, DEMO will delete individuals with one of the three methods including DEMO/parent、DEMO/closest/obj and DEMO/closest/dec. It can be concluded that the characteristic of multi-objective differential evolution algorithms in early research are as follow: (1) they are greedy in searching pareto optimal solutions (PS). (2) the introduction of pareto dominance extended the ability of algorithms from solving only single objective problems to MOP. (3) The principle of better solutions replacing original solutions are the power to push algorithms finding better solutions. However, issues like falling into local optimal, failing in solving complex problems are the difficulties that researchers facing at that time.

With the development of MODE, many operators and strategies are proposed for fixing the disadvantages of algorithms to improve their performance. Zheng et. al. developed a multi-objective differential evolution algorithm in 2008^12^, the new principle is used to replace the crossover operation in the original DE for improving the diversity of the population. In 2010, a diversity-enhanced multi-objective differential evolution algorithm (DE-CMODE) which combines a diverse memory bank with contemporary populations to improve the diversity of offspring was proposed by Qu^13^. In 2011, an improved MODE with adaptive parameter control strategy to accelerate the convergence speed and obtain a better-distributed PF was proposed by Bi et al. in^14^. In 2012, Yong et al. proposed a multi-objective strength Pareto chaotic differential evolution algorithm^15^, which introduced the truncated crowding operation and chaos substitution operation based on the uniform crowding mechanism to improve the convergence performance of the algorithm. In 2013, Zhou et al. proposed an improved MODE with adaptive crossover rate to improve the diversity of Pareto solutions and quality of PF^16^. In 2014, inspired by particle swarm optimization (PSO), Bourennani et al. introduced the concept of leadership frontier in MODE to further improve the convergence accuracy of the algorithm^17^. Due to the advantages of obtaining satisfactory results with less cost, the rapidly developing MODE are widely applied to practical problems. Fang et al. added direction guidance to MODE and applied the algorithm to solve the cold rolling regulations in 2017^18^. In 2018, CDMODE proposed by Xu et al. in which the crowding distance is introduced to divide population is applied to the optimal scheduling problem of the blast furnace gas system^19^. In CDMODE, the crowding distance is introduced into the multi-objective differential evolution algorithm and the population is divided according to the crowding distance, and applied it to the optimal scheduling problem of the blast furnace gas system. In 2019, the decomposition-based multi-objective evolutionary algorithm MOEA/D was applied to the layout planning problem of expressway electric vehicle charging stations by Tian^20^. Zhan et.al. proposed a new multiple populations for multiple objectives, a large number of experiments shown that the proposed algorithm has some better performance^21^. A dynamic Taylor Kriging (DTK) is proposed by combination with a multi-objective differential evolution (MODE) algorithm in 2015^22^, which it used to a multi-objective optimization of engineering problem and it performs better. Siwakorn et.al. proposed an interval success history based adaptive multi-objective differential evolution (iSHAMODE) and its hybrid variant with the whale optimisation algorithm (iSHAMODE-WO) for the main TSS truss optimisation loop^23^. Yu et.al. developed a constrained multi-objective differential evolution algorithm with ranking mutation operator in 2022, results demonstrated that this algorithm find well-distributed Pareto front^24^. Tian Ye team design a hybrid algorithm is tailored for Large-scale multi-objective optimization problems (LSMOPs) based on differential evolution and a conjugate gradient method. Compared to state-of-the-art evolutionary algorithms, and classic algorithms, the proposed algorithm show better performance for solving benchmark and real-world LSMOPs^25^.

After years of development, MODE is proven a simple and effective algorithm for solving MOP. However, though many methods for improving MODE are proposed constantly, MODE is still facing difficulties: as mentioned above, the greedy characteristic of MODE make it easy to confuse local optimal and global optimal. So it is a significant issue that how to force the algorithm to jump out of the local optimal on the premise of maintaining the greedy characteristic. Besides, a fast convergence speed is also what people expect. To solve the above difficulties, an improved multi-objective differential evolution algorithm based on a decomposition strategy (MOEA/D/DEM) is proposed in this paper, which can improve the performance of DE effectively and be applied to practical problems. The main advantages of MOEA/D/DEM are as follows:

(1) The neighbor intimacy factor is added in the process of generating offspring to improve the diversity of the population.

(2) a new mutation operator is designed to improve the local search ability of the algorithm. The performance of the proposed algorithm is tested by using the 16 test functions (DTLZ1-7 and WFG1-9). Compared with other algorithms (SMSMOEA MOEA/D/D MOEA/D/DE IMMOEA/D NSGAII IBEA), the results reveal that MOEA/ D/DEM algorithm does have a better search ability.

(3) The proposed algorithm is applied to a multi-objective nutrition decision problem and compared with other 10 algorithms (CDMODE, NSDE, PDE, MOEA/D, MOEA/D_DRA, NSGA_II, I_NSGA_II, AMOPSO/D, MOPSOSS, PAES). The results show that the MOEA/D/DEM algorithm can achieve the best performance.

The remainder of the paper is as follows. The related fundamental work is introduced in Section “Multi objective optimization problems and related algorithms”. The MOEA/D/DEM is introduced and analyzed in Section “Improved multi objective differential evolution algorithm based on decomposition”. The experiment results and analysis on the conventional test function optimization problems are shown and discussed in Section “Simulation results and analysis”. The application of MOEA/D/DEM in multi-objective nutritional decision problem is presented in Section “Application of the MOEA/D/DEM algorithm in the multi objective nutrition decision”. The conclusion will be given in Section “Conclusions”.

## Multi-objective optimization problems and related algorithms

### Related concepts of multi-objective optimization problems

Without loss of generality, a minimum problem is taken as an example.

#### Definition 1

Multi-objective optimization problem^26^ is described as.

1 $$\mathop {\text{min }}\limits_{{\text{X}}} { }f = \left( {f_{1} ,f_{2} , \ldots \ldots , f_{M} } \right)^{T}$$

2 $$g_{i} \left( {\text{X}} \right) \le 0,{\text{ i}} = 1,2, \ldots \ldots ,{\text{m}};{ }$$

3 $$h_{j} \left( X \right) = 0, j = 1,2, \ldots \ldots , n;$$

where $X={({x}_{1},{x}_{2},\dots \dots ,{x}_{D})}^{T}\in\Omega \subset {R}^{D}$ is the decision vector; $f={({f}_{1},{f}_{2},\dots \dots , {f}_{M})}^{T}\in\Lambda \subset {R}^{M}$ is the objective vector. ${g}_{i}$ is the $m$-th inequality constraint and ${h}_{j}$ is the $j$-th equality constraints. All decision vectors constitute a known $D$-dimensional decision space $\Omega$, and all objective vectors constitute an unknown $M$-dimensional target space $\Lambda$.

Better solutions are judged based on the obtained value in single objective problems, While in MOP, they are judged by Pareto dominance, which is a kind of most commonly used preference relationship. The definition of Pareto dominance is as follows.

#### Definition 2

(*Pareto domination*) If the feasible solutions ${\mathrm{X}}_{1}\in\Omega$ and ${\mathrm{X}}_{2}\in\Omega$ satisfy both Eqs. (4) and (5), then ${X}_{1}$ is said to be Pareto dominated by ${X}_{2}$, which is written as ${X}_{1}\prec {X}_{2}$.

4 $$\forall i = 1,2, \ldots ,M,f_{i} \left( {X_{1} } \right) \le f_{i} \left( {X_{2} } \right)$$

5 $$\exists j = 1,2, \ldots ,M, f_{j} \left( {X_{1} } \right) < f_{j} \left( {X_{2} } \right)$$

#### Definition 3

(*Pareto optimal solution*) If the feasible solutions ${\mathrm{X}}^{*}\in\Omega$ and $\mathrm{X}\in\Omega$ satisfy Eq. (6), then ${X}^{*}$ is names as the Pareto optimal solution.

6 $${\nexists }X \in {\Omega },X \prec X^{*}$$

#### Definition 4

(*Pareto optimal solution set*) The set of all Pareto optimal solutions for a multi-objective optimization problem is called the Pareto optimal solution set.

#### Definition 5

(*Pareto Front*) The front consists of points in objective space corresponding to the Pareto optimal solution set is called the Pareto front.

### Differential evolution algorithms

The first step of DE is initialization. The value of the $j$-th dimension of the $i$-th individual before iteration is set according to (7). During the iteration, the population of the $t$-th generation ${P}_{t}$ is described as (8).

7 $$x_{i,j}^{0} = x_{j,min} + rand\left( {0,1} \right){*}\left( {x_{j,max} - x_{j,min} } \right),j = 1,2, \ldots ,D$$

8 $$P_{t} = \left\{ {X_{i}^{t} { }|{ }X_{i}^{t} = \left( {x_{i,1}^{t} ,x_{i,2,}^{t} \ldots ,x_{i,j}^{t} \ldots ,x_{i,D}^{t} } \right)} \right\},{ }i = 1,2, \ldots ,{\text{N}},t = 1,2, \ldots ,T_{max}$$

where $D$ is the dimension of decision space; ${x}_{j,min}$ and ${x}_{j,max}$ represent the minimum and maximum values in the $j$-th dimension, respectively; $rand(\mathrm{0,1})$ is a random number in (0,1); $N$ is the population size and ${T}_{max}$ is the maximum number of iterations.

After the initialization, the mutation vector of each individual is obtained by (9) in the mutation operation, where ${X}_{{p}_{1}}^{t}$、${X}_{{p}_{2}}^{t}$、${X}_{{p}_{3}}^{t}$ are three individuals randomly chosen from population, and $i\ne p1\ne p2\ne p3$; F is the variation factor, which is different in many algorithms according to the purpose. The following crossover operation generates the test vector ${U}_{i}^{t}$ which inherits genes from parents and mutation vectors. The frequently used crossover operators are exponential crossover and binomial crossover. The final step of an iteration is selecting, which ensures the population size and pushes algorithm to evolve to a better optimality.

9 $$V_{i}^{t} = X_{{p_{1} }}^{t} + F\left( {X_{{p_{2} }}^{t} - X_{{p_{3} }}^{t} } \right)$$

### Multi-objective optimization algorithm based on a decomposition

A decomposition-based multi-objective evolutionary algorithm obtains a nondominated solution set by using the aggregation function to convert a multi-objective problem into many sub-problems, which are optimized simultaneously. A decomposition-based multi-objective evolutionary algorithm (MOEA/D) and the general framework is proposed by Zhang and Li in 2007 in^27^. The Chebyshev aggregation method and the minimization optimization problem are used as examples to introduce the general framework below.

#### Chebyshev aggregation method

$\lambda ={ {(\lambda }_{1}, \dots , {\lambda }_{i}, \dots {\lambda }_{N})}^{T}$ IS a set of weight vectors, where ${\lambda }_{i}=({\lambda }_{i,1} , \dots , {\lambda }_{i,M})$

10 $$\min g{(}x {|} \lambda_{i} , z^{*} ) = \mathop {\max }\limits_{1 \le m \le M} \left\{ {\lambda_{i,m} \left| {f_{m} \left( x \right) - z_{m}^{*} } \right|} \right\}$$

$$z^{*} = \left( {z_{1}^{*} , \ldots , z_{M}^{*} } \right)$$

11 $$z_{m}^{*} = \min \{ f_{m} \left( x \right) | x \in \Omega \} ,m = 1, \ldots ,M$$

where *x* represents a feasible solution, ${z}^{*}$ is a set of reference points, which often consist of the minimum objective function values currently achieving $.$

#### General framework for multi-objective optimization algorithms based on decomposition

1. Initialization

- Initialize algorithm parameters such as population size N.
- Set a set of weight vector $\lambda ={ {(\lambda }_{1}, \dots , {\lambda }_{i}, \dots {\lambda }_{N})}^{T}$. The sub-problems corresponding to nearby weight vectors usually have a relatively close relationship, so the Euclidean distance between every two weight vectors are calculated, and for each weight vector, select T closest weight vectors as neighbors. A set $B\left(i\right)=\{{i}_{1}, \dots , {i}_{T}\}$ is defined to preserve the neighbor of the $i$-th solution.
- Initialize the population and calculate the objective function value.
- Set the external archive EP = ∅ to store the nondominated solutions in population after each iteration.

(2) Updating

For each interation

- Use the corresponding neighbor to select the parent $x$ to generate the child $x^{\prime}$;
- Calculate the objective function value of $x^{\prime}$ and update the reference point set ${z}^{*}$. For each $m (1\le m\le M)$, ${z}_{m}^{*}={f}_{m}\left({x}^{\prime}\right)$ if ${f}_{m}\left({x}^{{\prime}}\right)\le {z}_{m}^{*}$.
- Update neighbor solution: for each $k\epsilon B\left(i\right),1\le k\le N$, if $g\left(x^{\prime} \right| {\lambda }_{k}, {z}^{*})\le g\left({x}_{k} \right| {\lambda }_{k}, {z}^{*})$, ${x}_{k}$ = $x^{\prime}$. For each $m (1\le m\le M)$, $f\_m(x\_k)=f\_m(x^{\prime})$;
- Update external archive EP: add $x^{\prime}$ to EP if $x^{\prime}$ is not dominated by individuals in EP and delete individuals dominated by $x^{\prime}$;
- Stop, if the max generation or max function evaluation is met, stop the program and output EP, otherwise skip to step (2).

## Improved multi-objective differential evolution algorithm based on decomposition

As said in section I, MODE suffers from the trap of local optimization, which limits it to achieve the Pareto optimal frontier. To solve this problem, an improved decomposition-based multi-objective differential evolution algorithm call MOEA/D/DEM is proposed in this paper. In MOEA/D/DEM, a neighbor intimacy factor for improving the searching ability to help the algorithm jump out of the local optimum is introduced. Moreover, after the study of many mutation methods, a new mutation method is proposed, in which the mutation vector generated by the original MODE may be modified with a certain probability. The proposed mutation method helps to enhance the local search ability which avoids individuals falling into the Pareto local optimum and accelerate the convergence speed. In the meantime, the external archive strategy is applied to preserve the nondominated solutions generated during the iteration process. In the rest of this section, we first introduce the neighbor intimacy operation and Gaussian mutation with variable-step, then a general framework of MOEA/D/DEM will be given.

### Neighbor intimacy operation

In the multi-objective differential evolution algorithm, the selection of parents has an important effect on the performance of the algorithm. As the iteration number increases, the diversity of the population and the alternative parents will decrease. In this paper, the neighbor intimacy factor NI is added in the process of parents selecting, and a reasonable method is chosen to select the neighbor. If a random value ${r}_{1}$(${r}_{1}\in \left[\mathrm{0,1}\right]$) is less than $NI$, parents are selected from the neighbor of the current individual; Otherwise, the parents are selected randomly from the neighbor of other individuals. $NI$ is a value that changes with time. In order to facilitate the comparison with other algorithms, $NI$ is fixed as a more reasonable value $(NI=0.8)$.

### Gaussian mutation strategy with variable step size

Mutation strategy plays a key role in MODE which has a significant effect for the performance of algorithm. Common mutation strategies^28^ include uniform mutation, non-uniform mutation, Gaussian mutation, Cauchy mutation and polynomial mutation, which aim to exploit new domain, find new search direction and escape limited data space.

The selection of suitable mutation step size is vitally important to the performance of the algorithm. A large step size means a faster convergence speed at the expense of the quality of the obtained PF while a small step size brings high convergence accuracy and slow convergence speed. In order to balance the contradiction between convergence speed and convergence accuracy in the condition of a fixed step size, a Gaussian mutation strategy with variable step size is proposed in this paper. Gaussian mutation strategy with variable step size can adjust the step size according to the different iterative environment. The proposed method balances the convergence speed and accuracy of the algorithm by shrinking step size as the iteration increases, which accelerates the convergence speed, improves the convergence accuracy and enhances the local searching ability of the algorithm. The description of Gaussian mutation strategy with variable step size is shown in (12) and (13):

12 $$x_{i,j}^{t} = x_{i,j}^{t} + N\left( {0,\sigma } \right)$$

13 $$\sigma = \left\{ {\begin{array}{*{20}c} {{-\!\!-}\frac{{x_{i,j}^{t} - x_{j,min} }}{{2 + c*\left( \frac{t}{T} \right)^{b} }} ,r = 0} \\ {\frac{{x_{j,max - } x_{i,j}^{t} }}{{2 + c*\left( \frac{t}{T} \right)^{b} }} ,r = 1} \\ \end{array} } \right.$$

where ${x}_{i,j}^{t}$ represents the $j$-th dimension of the $i$-th particle in the $t$-th iteration, $N(0,\sigma )$ represents the one-dimensional normal distribution random number with the mean value of 0, the variance is the mutation step size $\sigma$. $t$ is the current iteration number and $T$ is the maximum number of iterations. Parameters $b$ and $c$ are two control parameters, in which *c* controls the range of the mutation step while b controls the speed of the change of mutation along the number of iterations. $r$ is randomly selected in (0,1).

### The MOEA/D/DEM algorithm

**Figure 1:**
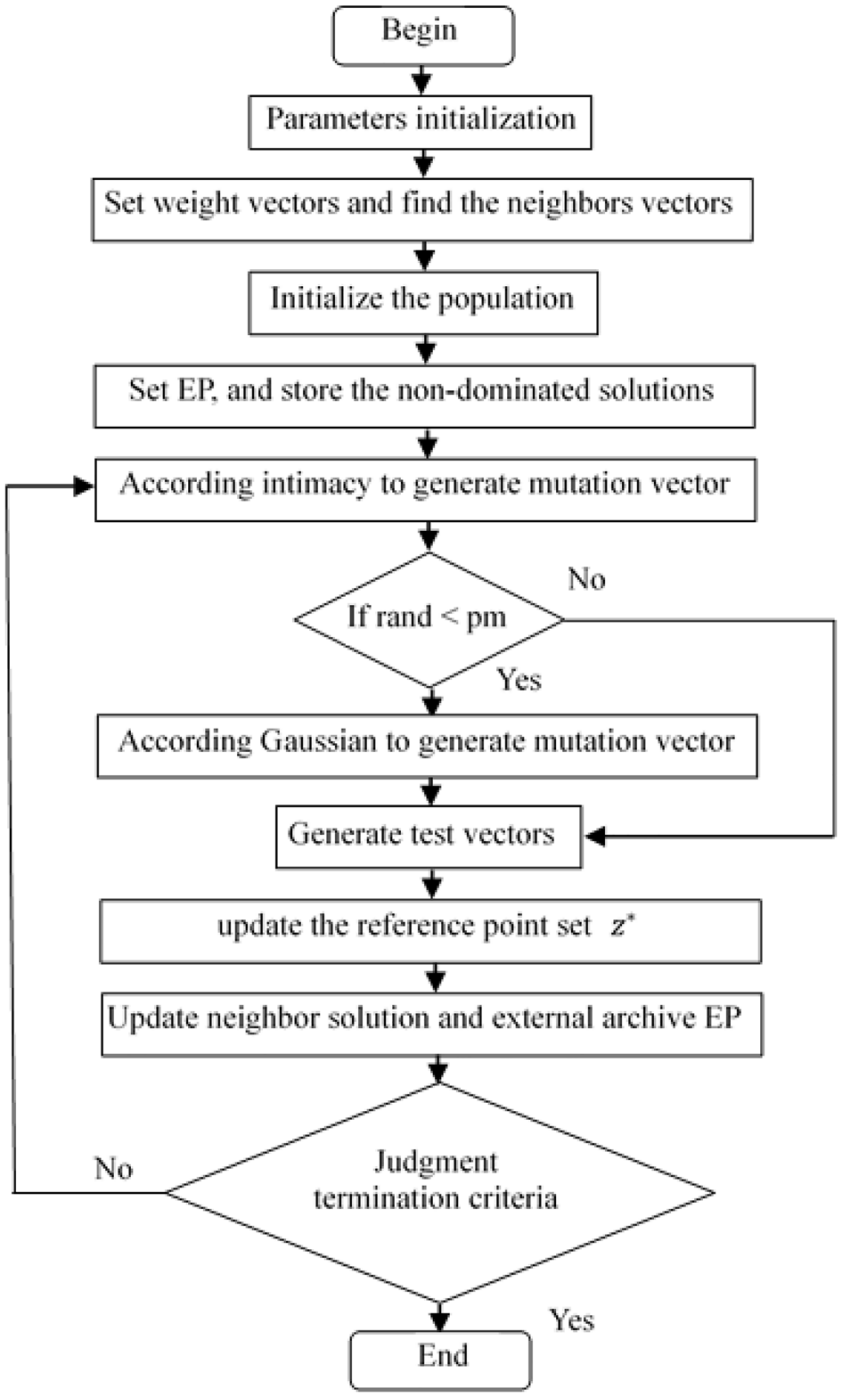
Flow chart of the MOEA/D/DEM algorithm.

In the MOEA/D/DEM, the solution generation strategy in GA is replaced by that in DE, and the neighbor intimacy factor is added. When the neighbor intimacy is satisfied, parents are selected from the neighbors of the current individual, otherwise the parents are randomly selected from the neighbors of other individuals. After generating the test vector in DE, the variable step size Gaussian mutation is carried out with a certain probability. In each iteration, the generated nondominated solutions are stored in external archive. The flow chart of the MOEA/D/DEM algorithm is shown in Fig. 1. The steps of the MOEA/D/DEM algorithm are as follows:

1. Initialization

- Initialize algorithm parameters, population size *N*, scaling factor *F*, crossover probability *CR*, neighbor intimacy *NI*, and variable step Gaussian distribution control parameters *b* and *c*.
- Set a set of weight vector $\lambda ={ {(\lambda }_{1}, \dots , {\lambda }_{i}, \dots {\lambda }_{N})}^{T}$. The sub-problems corresponding to nearby weight vectors usually have a relatively close relationship, so the Euclidean distance between every two weight vectors are calculated, and for each weight vector, select T closest weight vectors as neighbors. A set $B\left(i\right)=\{{i}_{1}, \dots , {i}_{T}\}$ is defined to preserve the neighbor of the $i$-th solution.
- Initialize the population and calculate the objective function value.
- Set the external archive EP = ∅ to store the nondominated solutions in population after each iteration.

(2) Update

For each i (1 ≤ i ≤ N).

If ${r}_{1}\le NI$ selects the parent from B(i), otherwise selects the parent from B(⌈rand*N⌉), and generates the mutation vector ${v}_{i}$ through the mutation operation;

Perform variable step size Gaussian mutation on the test vector ${v}_{i}$ to obtain the mutated test vector ${v}_{i}^{{{\prime}}}$;

Perform crossover operation to generate test vector ${u}_{i}$;

Calculate the objective function value of ${u}_{i}^{{{\prime}}}$ and update the reference point ${z}^{*}$. For each $m (1\le m\le M)$, if ${f}_{m}\left({u}_{i}^{{{\prime}}}\right)\le {z}_{m}^{*}$, then ${z}_{m}^{*}={f}_{m}\left({x}^{{{\prime}}}\right)$;

Update of neighbor solution: For each $j\epsilon B\left(i\right),1\le j\le N$, if $g\left({u}_{i}^{{{\prime}}}\right| {\lambda }_{j}, {z}^{*})\le g\left({x}_{j} \right| {\lambda }_{j}, {z}^{*})$, then ${x}_{j}={u}_{i}^{{{\prime}}}$, for each $m(1\le m\le M)$, ${f}_{m}$(${x}_{j}$)$={f}_{m}$(${u}_{i}^{{{\prime}}}$);

Update external archive EP: add $x^{{\prime}}$ to EP if $x{^{\prime}}$ is not dominated by individuals in EP and delete individuals dominated by $x{^{\prime}}$;

(3) Stop, if the max generation or max function evaluation is met, stop the program and output EP, otherwise skip to step (2).

## Simulation results and analysis

To illustrate the performance of MOEA/D/DEM in solving MOP, the study compared MOEA/D/DEM with six algorithms including SMSEMOA, MOEA/D/D, MOEA/D/DE, IMMOEA/D, NSGAII and IBEA in test suits DTLZ1-7 and WFG1-9. The population sizes of other algorithms are set to 100 while MOEA/D/DEM is 351. The maximum function evaluation to all algorithms are set to 25,000 and each problem is carried out 30 times independently. Part of the experiments are carried out in evolution multi-objective optimization platform(platEMO) which is developed by Tian et al.^29^. Parameters of all compared algorithms are the default value in platEMO.

Tables 1 and 2 are the IGD results of comparing MOEA/D/DEM with six other MOEAs in totally 16 problems. As shown in tables, MOEA/D/DEM obtains best values of all algorithms in DTLZ2, DTLZ6, DTLZ7 and WFG4-7, and ranks the second place in WFG8 and 9. Specially, the PF of MOEA/D/DEM in DTLZ6 is greatly superior to other algorithms, meaning that MOEA/D/DEM obtains an almost true PF. The last rows of the tables represent the final results that MOEA/D/DEM defeats or loses to other algorithms in 16 test problems. What can be obviously seen is that MOEA/D/DEM defeats SMSEMOA、MOEA/D/D、MOEA/D/DE、IMMOEA/D、IBEA in an overwhelming degree, and for NSGAII, MOEA/D/DEM defeats the algorithm in more than half of the problems. The aforementioned description indicating MOEA/D/DEM have a better performance comparing to other algorithms, implies that it can solve most problems in an outstanding level. It is worth mentioning that the compared algorithms include all the three types of methods to solve MOP (based on indicator, Pareto domination and decomposition), so the final result proves that MOEA/D/DEM can perform prominently in the comparison with all types of algorithms.

**Table 1:** IGD values of 7 algorithms in 16 problems.

Problem	M	D	SMSEMOA	MOEA/D/D	MOEA/D/DE	MOEA/D/DEM
DTLZ1	3	7	3.2336e-2 (5.53e-3) -	**2.1212e-2 (1.17e-3) -**	1.6425e-1 (3.02e-1) +	1.215e-1 (1.08e-2)
DTLZ2	3	12	7.9607e-2 (3.30e-3) +	5.4487e-2 (5.12e-5) +	7.6194e-2 (9.18e-4) +	**3.7589e-2 (3.97e-3)**
DTLZ3	3	12	7.9567e-1 (9.51e-1) -	1.9309e + 0 (8.99e-1) -	9.7194e + 0 (1.42e + 1) +	7.4991e + 0 (3.74e + 0)
DTLZ4	3	12	2.9013e-1 (2.82e-1) +	8.7125e-2 (1.24e-1) +	1.3456e-1 (7.35e-2) +	7.2056e-2 (1.69e-2)
DTLZ5	3	12	1.3882e-2 (1.97e-3) +	3.1319e-2 (1.20e-3) +	1.4331e-2 (1.58e-4) +	1.2417e-2 (2.08e-3)
DTLZ6	3	12	1.9727e-2 (6.73e-3) +	3.3802e-2 (1.11e-3) +	1.4486e-2 (4.15e-5) +	**5.3710e-5 (5.30e-6)**
DTLZ7	3	22	1.0759e-1 (9.36e-2) +	6.1928e-1 (2.64e-1) +	2.2600e-1 (1.19e-1) +	**1.9101e-2 (2.22e-3)**
WFG1	3	12	3.2299e-1 (3.76e-2) -	5.2582e-1 (1.11e-1) -	1.2850e + 0 (8.77e-2) -	1.3938e + 0 (9.50e-2)
WFG2	3	12	2.2422e-1 (1.55e-2) -	**1.8173e-1 (4.41e-3) -**	3.3720e-1 (1.69e-2) +	2.7596e-1 (2.60e-2)
WFG3	3	12	**9.6087e-2 (7.19e-3) -**	2.7549e-1 (1.15e-1) +	1.9132e-1 (4.24e-2) -	2.5722e-1 (3.68e-2)
WFG4	3	12	2.9898e-1 (1.50e-2) +	2.4269e-1 (1.82e-3) +	3.8948e-1 (9.95e-3) +	**2.2447e-1 (2.43e-2)**
WFG5	3	12	2.9919e-1 (1.55e-2) +	2.4763e-1 (3.06e-3) +	3.3703e-1 (3.51e-3) +	**8.1835e-2 (7.18e-4)**
WFG6	3	12	3.4065e-1 (2.20e-2) +	2.6886e-1 (3.03e-2) +	4.0963e-1 (3.38e-2) +	**2.5955e-1 (1.64e-4)**
WFG7	3	12	3.4525e-1 (2.09e-2) +	2.4615e-1 (2.08e-3) +	3.6272e-1 (5.53e-3) +	**1.9519e-1 (1.92e-2)**
WFG8	3	12	3.9012e-1 (1.30e-2) +	**3.0736e-1 (4.86e-3) -**	4.4277e-1 (2.36e-2) +	3.3046e-1 (1.73e-2)
WFG9	3	12	2.8153e-1 (1.37e-2) +	**2.4270e-1 (4.51e-3) -**	3.3375e-1 (4.68e-3) +	2.6565e-1 (1.33e-2)
+ /−/ =			11/5/0	10/6/0	14/2/0	

The best of the comparison results are in [bold].

**Table 2:** IGD values of 7 algorithms in 16 problems.

Problem	M	D	NSGAII	IMMOEA/D	IBEA	MOEA/D/DEM
DTLZ1	3	7	6.6113e-2 (9.36e-2) -	2.0488e + 0 (7.26e-1) +	1.6283e-1 (2.47e-2) +	1.215e-1 (1.08e-2)
DTLZ2	3	12	6.8715e-2 (2.45e-3) +	7.5561e-2 (7.24e-4) +	8.0053e-2 (2.68e-3) +	**3.7589e-2 (3.97e-3)**
DTLZ3	3	12	**6.3928e-1 (6.48e-1) -**	3.3142e + 1 (7.64e + 0) +	6.5517e-1 (3.44e-1)-	7.4991e + 0 (3.74e + 0)
DTLZ4	3	12	**6.7970e-2 (2.60e-3) -**	1.1673e-1 (8.14e-2) +	8.0213e-2 (3.29e-3) +	7.2056e-2 (1.69e-2)
DTLZ5	3	12	**5.7981e-3 (3.62e-4) -**	2.7384e-2 (4.24e-4) +	1.5817e-2 (1.43e-3) +	1.2417e-2 (2.08e-3)
DTLZ6	3	12	5.9233e-3 (3.35e-4) +	3.6987e + 0 (1.53e-1) +	2.6295e-2 (4.10e-3) +	**5.3710e-5 (5.30e-6)**
DTLZ7	3	22	1.0396e-1 (8.41e-2) +	1.9864e-1 (1.10e-2) +	1.2013e-1 (1.58e-1) +	**1.9101e-2 (2.22e-3)**
WFG1	3	12	2.4273e-1 (2.60e-2) -	1.1454e + 0 (8.10e-2) -	**1.8924e-1 (8.96e-3)-**	1.3938e + 0 (9.50e-2)
WFG2	3	12	2.2243e-1 (1.11e-2) -	3.2846e-1 (7.25e-3) +	2.9585e-1 (6.00e-3) +	2.7596e-1 (2.60e-2)
WFG3	3	12	1.0564e-1 (1.48e-2) -	2.2580e-1 (1.47e-2) -	3.8814e-2 (1.24e-3)-	2.5722e-1 (3.68e-2)
WFG4	3	12	2.6984e-1 (1.08e-2) +	3.5715e-1 (4.64e-3) +	3.1826e-1 (1.23e-2) +	**2.2447e-1 (2.43e-2)**
WFG5	3	12	2.7941e-1 (7.96e-3) +	3.5003e-1 (9.95e-3) +	3.2148e-1 (1.32e-2) +	**8.1835e-2 (7.18e-4)**
WFG6	3	12	3.1178e-1 (1.98e-2) +	3.6400e-1 (1.22e-2) +	3.4018e-1 (1.25e-2) +	**2.5955e-1 (1.64e-4)**
WFG7	3	12	2.8129e-1 (1.26e-2) +	3.6202e-1 (5.52e-3) +	3.2668e-1 (1.76e-2) +	**1.9519e-1 (1.92e-2)**
WFG8	3	12	3.6479e-1 (9.18e-3) +	3.9937e-1 (6.67e-3) +	3.3979e-1 (9.43e-3) +	3.3046e-1 (1.73e-2)
WFG9	3	12	2.7765e-1 (1.14e-2) +	3.4361e-1 (7.66e-3) +	2.9376e-1 (9.94e-3) +	2.6565e-1 (1.33e-2)
+ /-/ =			9/7/0	14/2/0	13/3/0	

The best of the comparison results are in [bold].

In the comparison of best values, MOEA/D/DEM obtains best values in six problems, while NSGAII and MOEADD obtains best values in three and four problems respectively, and SMSEMOA and IBEA obtain only one best value. In the problems that best values are obtains by MOEA/D/DEM, DLTZ6 has a puzzling PF because the distance of true PF and the dominated PF is close; DTLZ7 has a discontinuous PF^30^; WFG4 and 5 are multimodal and deceptive, respectively^31^. So MOEA/D/DEM can acquire satisfactory results in many types of problems. It can be concluded from the above content that MOEA/D/DEM have the best comprehensive capability comparing to other algorithms, that is to say, it is suitable to solve many types of problems. This means MOEA/D/DEM is competitive to solve practical problems since the characteristics of real-world problems are always unclear.

**Figure 2:**
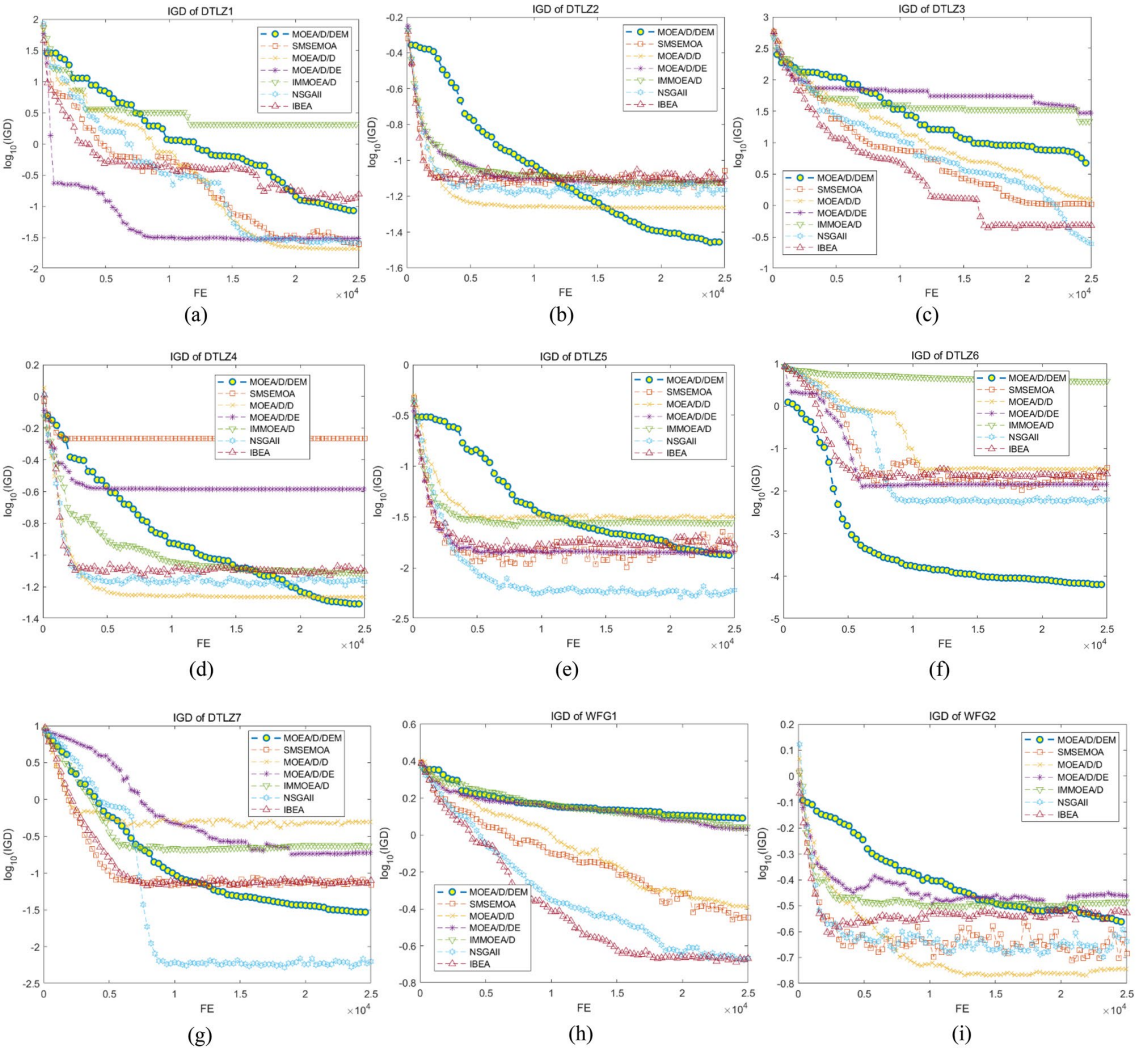

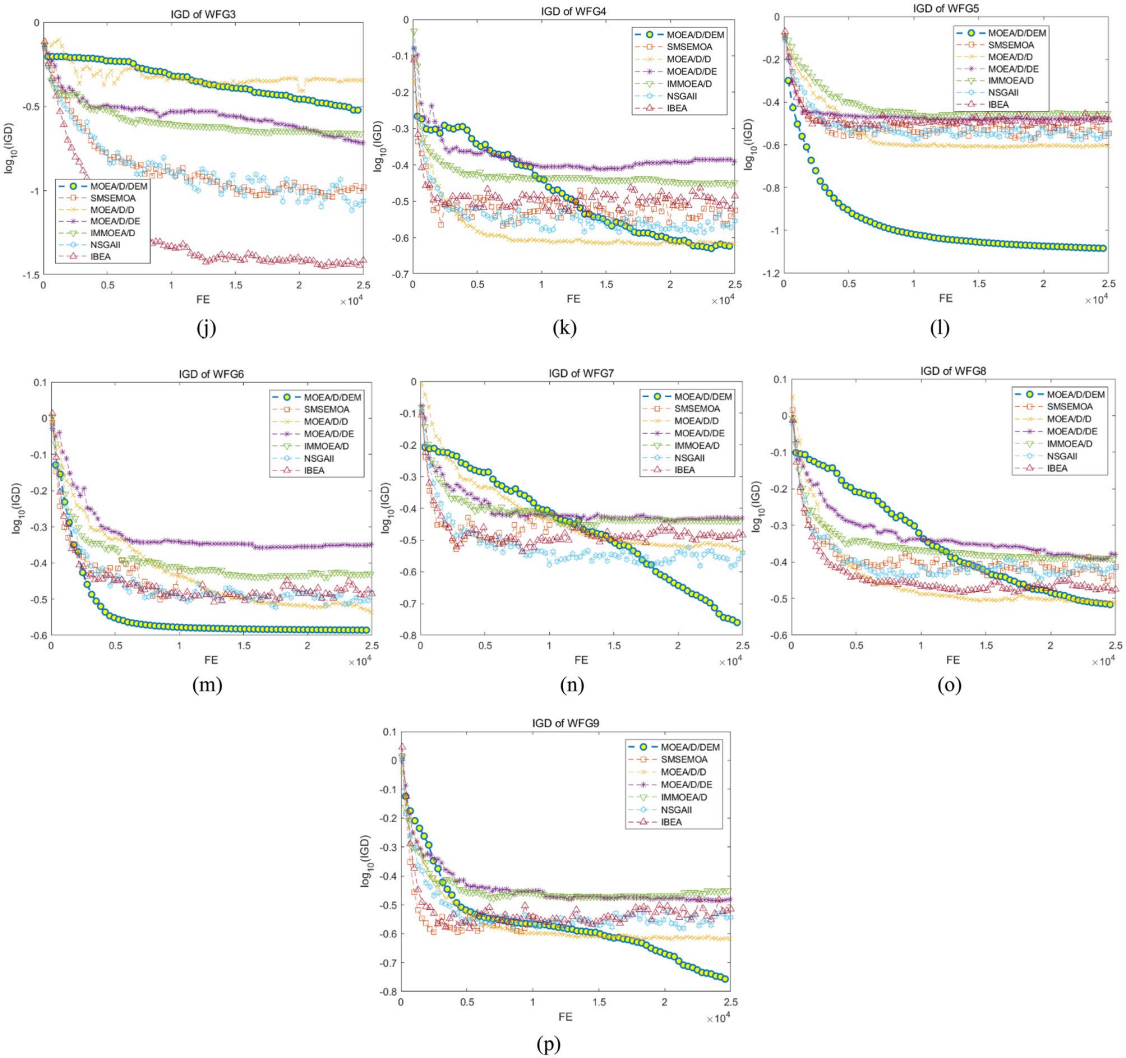
The change of IGD value of all algorithms in 16 problems.

Figure 2 show the change of IGD values of all algorithms as the function evaluation increases. The figures clearly represent that MOEA/D/DEM performs best in more than half of problems. Moreover, though MOEA/D/DEM gets the best in DTLZ2, DTLZ4, WFG4, WFG7-9, the slope of the IGD value curve still has an obvious down trend when it meets the maximum function evaluation, which means the algorithm has a tendency to search for a better PF, proving that MOEA/D/DEM has a superior searching ability. This owes to the use of the neighbor intimacy factor, with which the parents may be chosen in other individuals’ neighbors with a certain probability, giving chance to individuals to jump out of the local optimal solutions and obtain a better-distributed PF. In addition, due to the difference of population size, the iteration of MOEA/D/DEM is much less than other algorithms. This indicates MOEA/D/DEM has a satisfactory convergence speed because it acquires a better PF with less iterations. This improvement is brought by the use of proposed Gaussian mutation strategy. The step size of mutation gradually shrinks as the iteration increases, helping the algorithm quickly search for optimal solutions in early process while ensuring the convergence speed and the quality of PF by avoiding the influence of the excessive step size in the later stage.

In conclusion, MOEA/D/DEM performs outstanding in the comparison with other algorithms, confirming it is a preeminent MOEA with a good convergence speed which can obtain satisfactory PF. Besides, Due to the introduction of neighbor intimacy factor and special Gaussian mutation strategy, the convergence speed and quality of PF of MOEA/D/DEM are further improved. Moreover, MOEA/D/DEM performs prominently in many types of problems, meaning it is suitable to solve practical problems. To further verify the performance of MOEA/D/DEM in solving real-world problems, it is applied to solve a nutrition decision problem, in which the results still demonstrate the competitiveness of MOEA/D/DEM. This is introduced at length in the following part of the paper.

## Application of the MOEA/D/DEM algorithm in the multi-objective nutrition decision

### Multi-objective nutrition decision problem

#### Optimization objects

The nutrients that human need are various in real life. In this paper, MOEA/D/DEM is applied to a three-objective nutrition decision problem. Taking energy, protein and carbohydrate as an example, the objects for optimization are the closeness of the actual intake of the three nutrients and the recommended value. Based on the provided recommended value of the daily nutrient intake of an adult man in^32^ and assuming the nutrition intake ratios for three meals (breakfast, lunch and dinner) are 30%, 40% and 30% receptively, the recommended value of nutrient intake for lunch can be acquired, which is described as $B=\left({b}_{1},{b}_{2},\dots ,{b}_{m}\right).$

#### The optimization model

Referring to the multi-objective nutrition decision optimization model in^33^, the mathematical model in this paper is established as (14). According to the recommended nutrient intake of a an adult man in lunch $B=\left({b}_{1},{b}_{2},\dots ,{b}_{m}\right)$, a suitable nutrition plan $F(X)=\left({f}_{1}(X),{f}_{2}(X),\dots ,{f}_{m}(X)\right)$ which consist of $n$ kinds of food should be as close to B as possible. For the balance of nutrients, the intake of each kind of food except oil should be greater than 0 kg and less than 1.5 kg, while the intake of oil should be control in (0.01, 0. 05) kg every day.

14 $$\begin{array}{*{20}l} {\left\{ {\begin{array}{*{20}l} {\min F\left( X \right) = \left( {{ }f_{1} \left( X \right),f_{2} \left( X \right), \ldots ,f_{m} \left( X \right)} \right)} \hfill \\ {f_{{\text{i}}} \left( X \right) = \frac{{\left| {a_{i1} x_{{1{ }}} + a_{i2} x_{{2{ }}} + \ldots + a_{in} x_{{n{ }}} - b_{m} } \right|}}{{b_{m} }} ,i = 1,2, \ldots ,m} \hfill \\ {s.t. 0 \le x_{{i{ }}} \le 1.5 ,i = 1,2, \ldots ,n - 1;} \hfill \\ { 0.01 \le x_{{n{ }}} \le 0.05} \hfill \\ \end{array} } \right.} \hfill \\ \end{array}$$

${a}_{i1},{a}_{i2},\dots , {a}_{in}$ in (14) represent the nutrient content of each kind of food and ${x}_{1},{x}_{2},\dots , {x}_{n}$ are decision variables representing the weight of the selected food.

### The experiments

The recommended value of the daily intake for an adult man with moderate labor intensity is shown in Table 3 below.

**Table 3:** Recommended intake for an adult man of the three nutrients.

Nutrients	Energy(kcal/d)	Protein(g/d)	Carbohydrate (g/d)
Recommended Intake	2600	65	120

Supposing the number of the available food is $n$=22, the contents of three nutrients in these foods are listed in Table 4, which is extracted from^34^. In the experiments, the maximum function evaluation is set to 10,000. The parameter settings of all algorithms are shown in Table 5.

**Table 4:** The value of three nutrients per 100 g of each kind of food.

Nutrients Food	Energy (kcal)	Protein (g)	Carbohydrate (g)
Rice	116	2.6	25.9
Noodles	284	8.3	61.9
Pork (fat and lean)	395	13.2	2.4
Beef (lean)	106	20.2	1.2
Lamb (lean)	118	20.5	0.2
Chicken breast	133	19.4	2.5
Egg	147	12.8	1.4
Duck eggs	180	12.6	3.1
Goose egg	196	11.1	2.8
Fish	113	16.6	0
Shrimp	83	16.6	0.8
Milk	54	3	3.4
Tofu	81	8.1	4.2
Tomato	19	0.9	4
Chinese cabbage	17	1.5	3.2
Mushroom	20	2.7	4.1
Carrot	40	1.2	9.5
Potato	76	2	17.2
Apple	52	0.2	13.5
Pear	44	0.4	13.5
Banana	91	1.4	22
Cooking oil	899	0	0

**Table 5:** Parameter Settings of all comparing algorithms.

Algorithms	Parameter settings
MOEA/D/DEM	Population size N = 351; number of neighbors T = 20; scaling factor F = 1; crossover probability CR = 0.5; mutation probability pm = 1/D (D is the number of decision variables); variable step size Gaussian mutation control parameter c = 4 , B = 0.5;
CDMODE	Population size N = 100; crossover probability CR = 0.3; sparse threshold e1 = 0.7; congestion threshold e2 = 0.3; external archive size Np = 100;
NSDE	Population size N = 100; maximum scaling factor Fmax = 0.9; minimum scaling factor Fmin = 0.4; minimum crossover probability CRmin = 0.3; maximum crossover probability CRmax = 0.8;
PDE	Population size N = 100;
NSGA_II	Population size N = 100; crossover probability pc = 1; distribution index ${\eta }_{c}$=15, ${\eta }_{m}$=20; mutation probability pm = 1/D (D is the number of decision variables);
AMOPSO/D	Population size N = 351; number of neighbors T = 30; mutation probability Pm = 0.5; maximum speed inertia Wmax = 1; minimum speed inertia Wmin = 0.35
PAES	Probability of mutation Pm = 0.5; distribution index ${\eta }_{m}$=20;
MOEA/D	Population size $N=351$; Number of neighbors $T=10$; Mutation probability $pm=1/D$($D$ is the number of decision variables); Distribution index ${\upeta }_{\mathrm{c}}=15$, ${\upeta }_{\mathrm{m}}=20$;
MOEA/D_DAR	Population size $\mathrm{N}=1000$; number of neighbors $\mathrm{T}=100$; Scaling factor $\mathrm{F}=0.5$; Crossover probability $\mathrm{CR}=1$; Distribution index ${\upeta }_{\mathrm{m}}=20$; Parent selection probability $\updelta$=0.9
I_NSGA_II	Population size *N* = 100; Crossover probability *Pc* = 0.95; Mutation probability $\mathrm{pm}=1/\mathrm{D}$($\mathrm{D}$ is the number of decision variables); Distribution index ${\upeta }_{\mathrm{c}}=15$, ${\upeta }_{\mathrm{m}}=20$;
MOPSOSS	Population size *N* = 5; Global optimal archive size *k* = 4; *RSS* = 4; Mutation probability $pm=1/D$($D$ is the number of decision variables); Velocity inertia $\mathrm{w}$=0.3; Learning factor *c1* = 0.1, *c2* = 1.4; Reference set size Maximum spread points *MSS* = 7; BLX operator parameters $\mathrm{\alpha }=0.5$;

### Experiment results

In order to demonstrate the competitiveness of MOEA/D/DEM in solving multi-objective nutrition decision problems, it is compared with other 10 algorithms including CDMODE, NSDE, PDE, MOEA/D, NSGA_II, PAES, MOEA/D_DRA, I_NSGA_II, AMOPSO/D and MOPSOSS (See Table 6). To evaluate the performance of all algorithms, statistics including the average number and nutrient compliance rate $CR$ of meal plans obtained in 10 independent experiments are used as indicators.

**Table 6:** The number of meal plan obtained by all algorithms.

Algorithms	MOEA/D/DEM	NSGAII	AMOPSO/D	CDMODE	I_NSGAII	MOEA_D
Number of meal plans	255	63	70	96	62	108

Algorithms	MOEA/D/DEM	MOEA_D_DRA	MOPSOSS	NSDE	PAES	PDE
Number of meal plans	255	9	110	73	143	38

The average number of meal plans obtained by MOEA/D/DEM and other 10 algorithms is shown in Table 6. It can be obviously seen that the number of meal plans obtained by MOEA/D/DEM is much more than that obtained by other algorithms. This means that MOEA/D/DEM provides more choices of meal plan compared with the other 10 algorithms, which benefits from the introduction of neighbor intimacy factor. The average nutrient compliance rate $CR$ of the meal plan obtained by 11 algorithms in 10 independent experiments is shown in Table 7. The compliance rate is defined as

15 $$CR = \frac{{actual\, nutrient\, intake}}{{ recommended\, nutrient\, intake}}$$

**Table 7:** The average nutrient compliance rate of the meal plan of all algorithms.

Algorithms	Average energy compliance rate (%)	Average protein compliance rate	Average carbohydrate compliance rate	Comprehensive average compliance rate
MOEA/D/DEM	97.61	**104.21**	106.27	**102.70**
NSGAII	96.14	114.79	108.11	106.35
AMOPSO/D	297.47	491.92	481.47	423.62
CDMODE	101.34	157.93	125.56	128.28
I_NSGAII	96.40	115.82	108.82	107.01
MOEA_D	96.56	109.08	114.84	106.83
MOEA_D_DRA	753.22	2315.40	1116.70	1395.11
MOPSOSS	86.55	131.45	182.10	133.37
NSDE	98.25	124.72	**101.99**	108.32
PAES	96.58	155.52	141.87	131.32
PDE	**98.78**	207.25	117.24	141.09

The best of the comparison results are in [bold].

$CR$ represents the average compliance rates of the three nutrients. It’s obvious that a better solution should have a $CR$ value closer to 100%. It can be seen from Table 7 that the best results in average energy, protein and carbohydrate compliance rate are obtained by PDE, MOEA/D/DEM and NSDE respectively. When respect to the comprehensive compliance rate which is obtained by averaging the $CR$ value of the three chosen nutrients, MOEA/D/DEM performed best. The results confirm that MOEA/D/DEM considers all objects comprehensively in solving MOP instead of considering only single object. This presents the capacity and practicability of MOEA/D/DEM as an MOEA. The reason of the superior performance is because the neighbor intimacy factor and the Gaussian mutation strategy with variable step size improve the exploitation ability of MOEA/D/DEM, avoiding the algorithm falling into the dilemma of Pareto domination once it finds the optimum in one object.

In summary, MOEA/D/DEM presented the best performance in solving multi-objective nutrition decision problem in the comparison with other 10 algorithms. The algorithm can search for Pareto optimal solutions comprehensively in decision space with a fast speed, which verified the outstanding ability of MOEA/D/DEM when solving practical MOP.

## Conclusions

A decomposition-based multi-objective differential evolution algorithm MOEA/D/DEM is proposed in this paper. The neighbor intimacy factor in MOEA/D/DEM algorithm increases the diversity of the population and prevents the population from falling into local optimum. The variable step size Gaussian mutation strategy enhances the local search capability and the convergence speed and accuracy of the algorithm. These two strategies enable MOEA/D/DEM algorithm to have higher convergence accuracy and faster convergence speed than other comparing algorithms, implying that MOEA/D/DEM can solve multi-objective optimization problems more effectively, which is verified by simulation experiments on test functions DTLZ1-7 and WFG1-9.

MOEA/D/DEM algorithm is applied to the three-objective nutrition decision problem in this paper and is compared with other 10 algorithms to further demonstrate the ability. The experimental results showed that MOEA/D/DEM obtained the most number of meal matching schemes among 11 algorithms and perform best in nutrient comprehensive compliance rate. The experiment proves that MOEA/D/DEM can solve the three-objective nutrition decision problem well, indicating MOEA/D/DEM is a competitive MOEA in solving practical problems.

To sum up, MOEA/D/DEM is a superior algorithm. However, an algorithm that using less cost to obtain a better PF with a faster convergence speed should always be the persistent goal of all researchers in multi-objective algorithm field. Our expectation is to develop more algorithms which can solve practical problems with a better performance.

## Supplementary Information

Supplementary Information.

## Data Availability

The datasets used and/or analyzed during the current study available from the corresponding author on reasonable request.
