# Supplementary material for: Improved multi-objective differential evolution algorithm based on a decomposition strategy for multi-objective optimization problems
Source: Sci Rep. 2022 Dec 7;12:21176. doi: 10.1038/s41598-022-25440-7 (PMC9729643; doi:10.1038/s41598-022-25440-7)
Supplement: Supplementary file 1 — Supplementary Information. [file 41598_2022_25440_MOESM1_ESM.doc]

**Appendix 1**

**Test functions**

**DTLZ1**


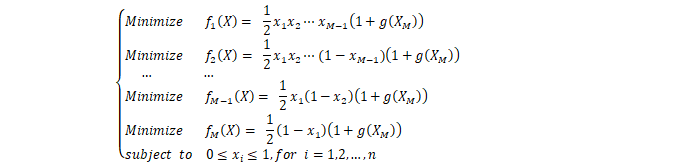


Among them,


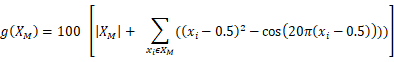


The true Pareto optimal frontier of DTLZ1:


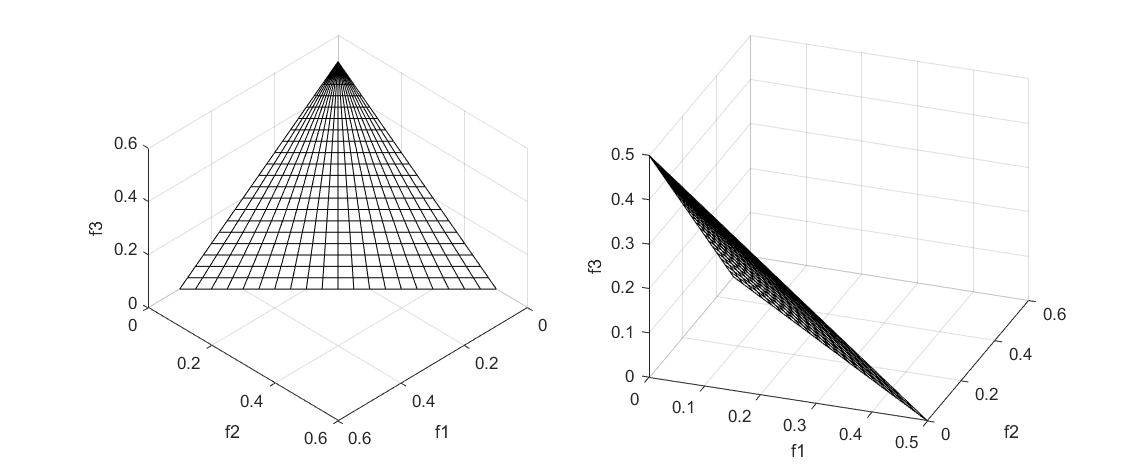


**DTLZ2**


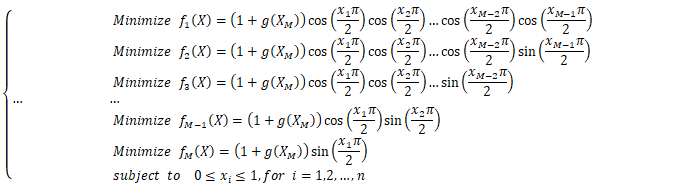


Among them,


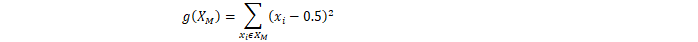


The true Pareto optimal frontier of DTLZ2:


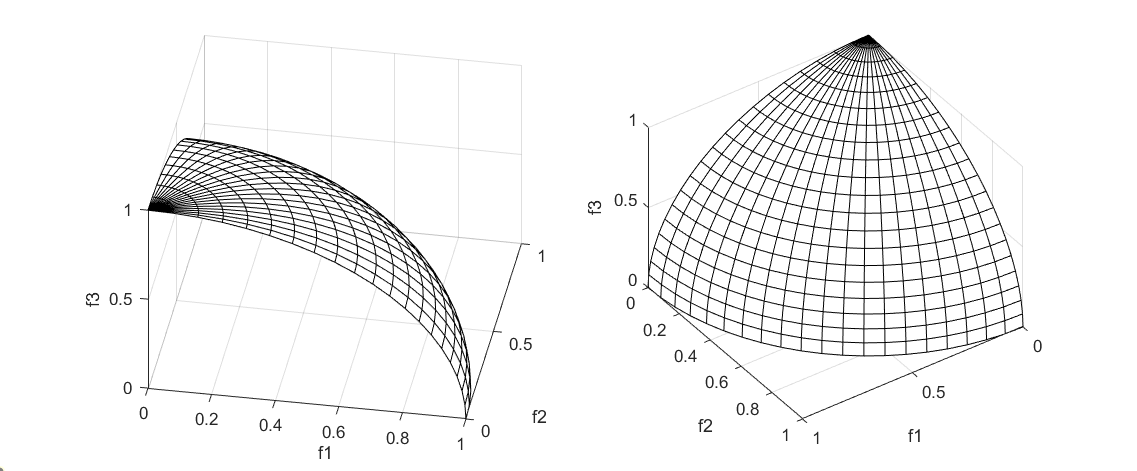


**DTLZ3**


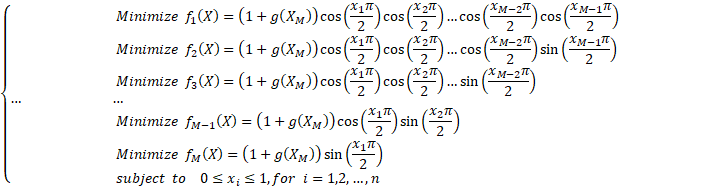


where,


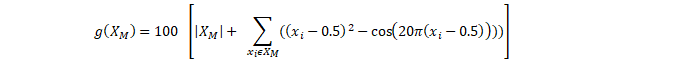


The true Pareto optimal frontier of DTLZ3:


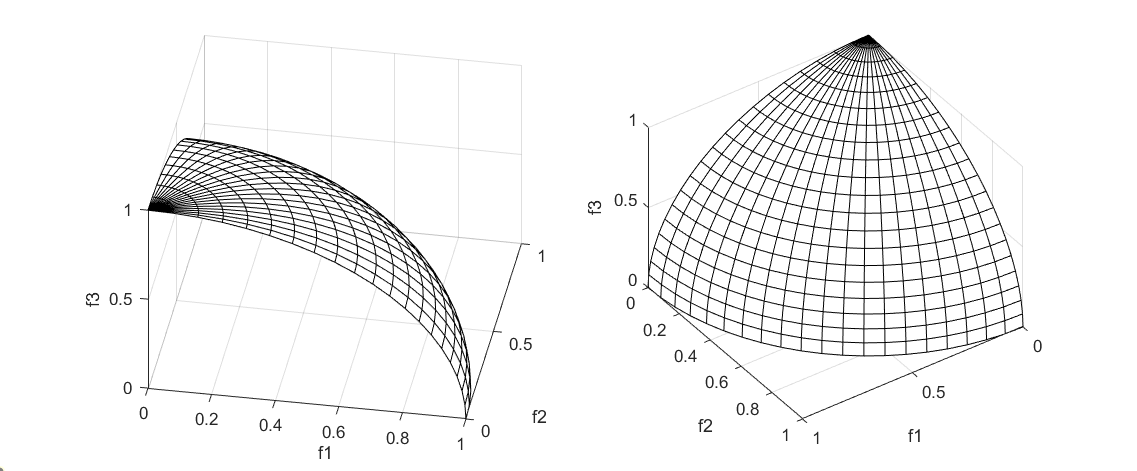


**DTLZ4**


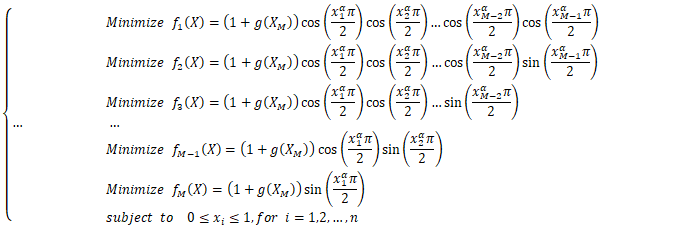


where,


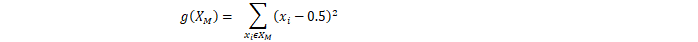


The true Pareto optimal frontier of DTLZ4:


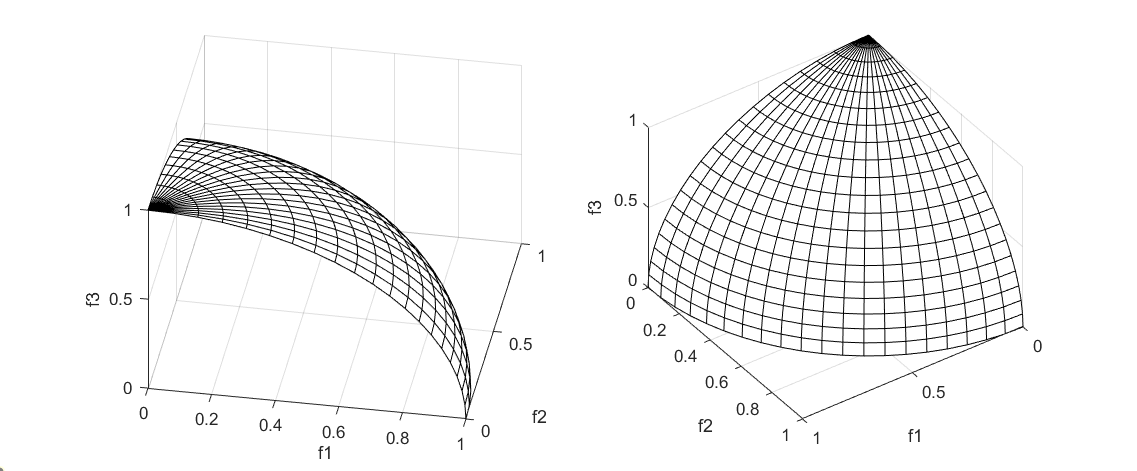


**DTLZ5**


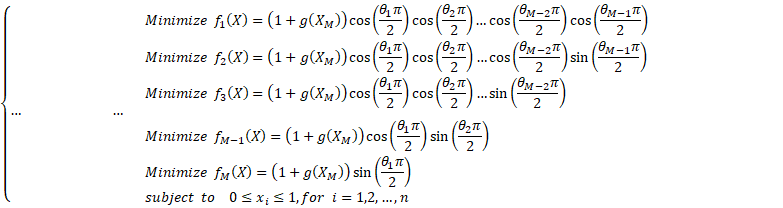


where,


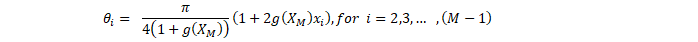


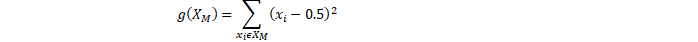


The true Pareto optimal frontier of DTLZ5:


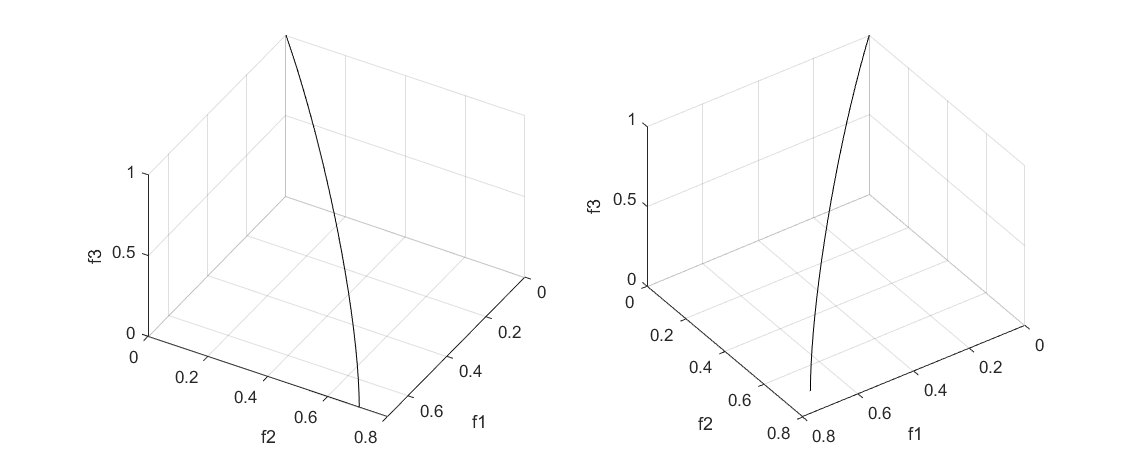


**DTLZ6**


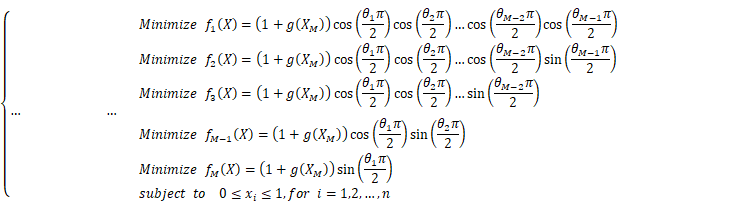


where,


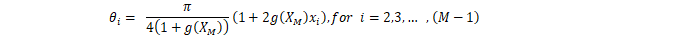


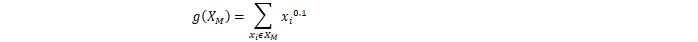


The true Pareto optimal frontier of DTLZ6:


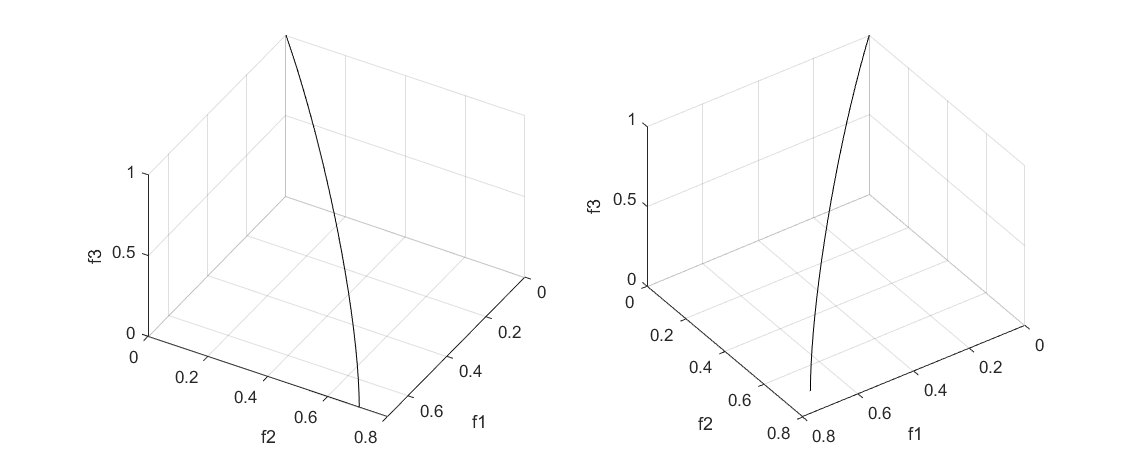


**DTLZ7**


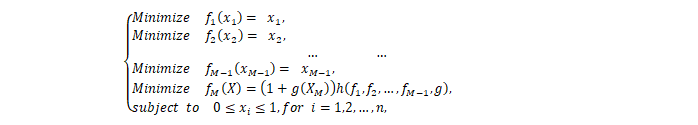


where,


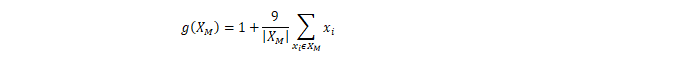


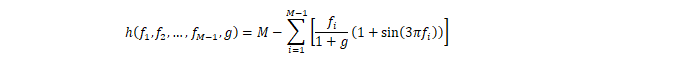


The true Pareto optimal frontier of DTLZ7:


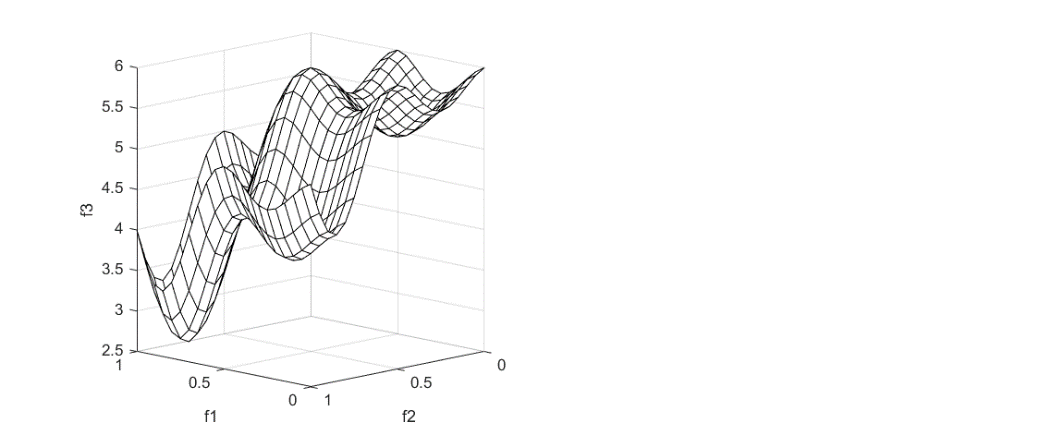

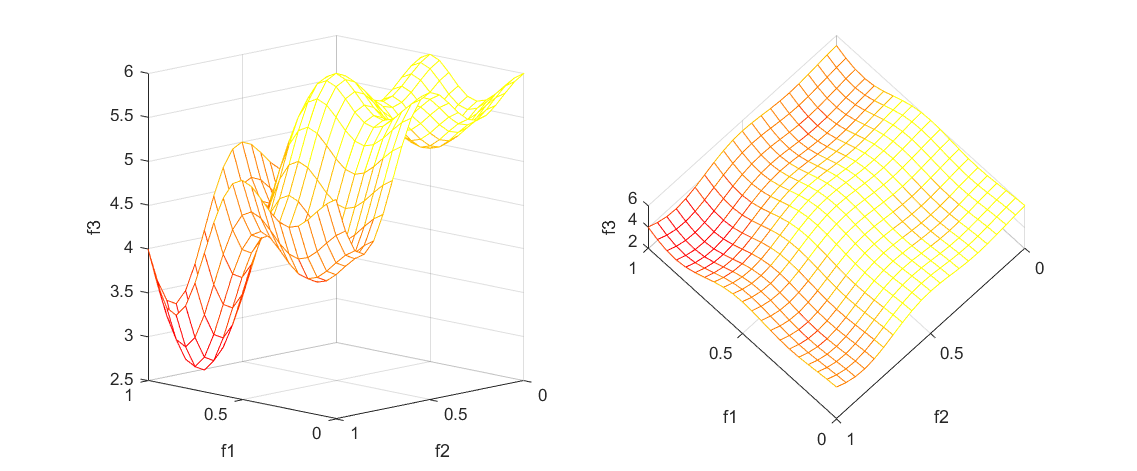


**Appendix 2**

**Calculation of anti-generation distance (IGD)**


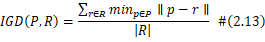


where P represents the population to be evaluated, R represents a set of reference points on the front of Pareto, |R| represents the total number of reference points, p-r represents the Euclidean distance from p to the reference point r in the target space. IGD measures the average distance from each reference point in R to the nearest solution in P. The smaller is the IGD value, the higher is the similarity between P and R.
